# Mammalian Diaphanous-Related Formin 1 Regulates GSK3β-Dependent Microtubule Dynamics Required for T Cell Migratory Polarization

**DOI:** 10.1371/journal.pone.0080500

**Published:** 2013-11-18

**Authors:** Baoxia Dong, Steven S. Zhang, Wen Gao, Haichun Su, Jun Chen, Fuzi Jin, Ajay Bhargava, Xiequn Chen, Lars Jorgensen, Arthur S. Alberts, Jinyi Zhang, Katherine A. Siminovitch

**Affiliations:** 1 Departments of Immunology and Molecular Genetics, University of Toronto, Mount Sinai Hospital, Samuel Lunenfeld Research Institute and Toronto General Research Institutes, Toronto, Ontario, Canada; 2 Department of Hematology, Xijing Hospital, Xian, Shaanxi, China; 3 Cell Structure and Signal Integration Laboratory, Van Andel Institute, Grand Rapids, Michigan, United States of America; 4 Department of Medicine, University of Toronto, Toronto, Ontario, Canada; King's College London, United Kingdom

## Abstract

The mammalian diaphanous-related formin (mDia1), a Rho-regulated cytoskeletal modulator, has been shown to promote T lymphocyte chemotaxis and interaction with antigen presenting cells, but the mechanisms underpinning mDia1 roles in these processes have not been defined. Here we show that mDia1^-/-^ T cells exhibit impaired lymphocyte function-associated antigen 1 (LFA-1)-mediated T cell adhesion, migration and in vivo trafficking. These defects are associated with impaired microtubule (MT) polarization and stabilization, altered MT dynamics and reduced peripheral clustering of the MT plus-end-protein, adenomatous polyposis coli (APC) in migrating T cells following LFA-1-engagement. Loss of mDia1 also leads to impaired inducible inactivation of the glycogen synthase kinase (GSK) 3β as well as hyperphosphorylation and reduced levels of APC in migrating T cells. These findings identify essential roles for the mDia1 formin in modulating GSK3β-dependent MT contributions to induction of T-cell polarity, adhesion and motility.

## Introduction

Immune homeostasis and adaptive immune responses depend upon the coordinated adhesion and migration of T cells which enables trafficking of both naïve and effector cells through the circulation and across secondary lymphoid organs or inflamed tissues [1]. These multistep processes are dependent on sequential activation of chemokine receptors and integrins through engagement with their ligands, enabling coordinated T-cell adhesion and motility during *in vivo* T-cell trafficking [2]. β2 integrin LFA-1 plays a particularly important role in modulating T cell adhesion and motility, its interaction with ICAM-1 (intercellular adhesion molecule 1) evoking T cell polarization and enabling T cells to adhere, crawl and thereby transmigrate across vascular endothelium [3,4]. T cell polarization is essential to these processes and requires extensive cytoskeletal remodeling that enables surface receptor, intracellular proteins and organelle redistribution so as to generate front-rear polarized morphology and forward protrusive forces driving directional migration [2]. Microtubule (MT) dynamics play integral roles in the morphologic rearrangement underpinning T cell migratory polarity, migration of these cells associated with reorientation of the microtubule organizing centre (MTOC) and posterior displacement of the microtubular array so as to generate an adhesive uropod that stabilizes cell position [5-7]. MT dynamics appear to influence not only such asymmetric T cell activities as adhesion and directional migration, but also T cell-dendritic cell contact, intracellular transport and other polarity-dependent processes critical to T cell motility and activation [8-11].

Although MT rearrangement is integrally involved in T cell polarization, the molecular pathways linking MT dynamics to specific T cell responses are poorly understood. In recent years, the mammalian diaphanous-related formin mDia1 has emerged as a key regulator of actin polymerization in haemopoietic cells, its activity mediated primarily via its FH2 domain and induced by interaction with activated Rho GTPase and consequent release from autoinhibitory structural constraints [12,13]. One of three members of the mDia formin subfamily, mDia1 is the prominent mDia expressed in T cells and has been implicated in T cell antigen receptor (TCR)-driven proliferative as well as chemokine-evoked migratory responses [14,15]. In addition to facilitating many actin-driven cell processes, mDia1 has also been implicated in reorientation of the MTOC downstream of TCR engagement in cytotoxic T cells and its upstream effector, Rho, has been shown to regulate chemokine-driven T cell cytoskeletal polarization [16,17]. These data suggest mDia1 involvement in the MT dynamics that enable T cells to polarize and engage in the adhesive and migratory responses underpinning T cell trafficking. To further define the influence of mDia1on MT-dependent T cell polarizing responses, we investigated mDia1’s contributions to MT dynamics associated with LFA-1-driven T cell migratory polarization. Here we show that the acquisition of polarized morphology and adhesion/transmigration consequent to cell contact with ICAM-1, as well as the ability to traffic through lymph nodes and to inflammatory sites *in vivo*, are profoundly impaired in T cells from mDia1^-/-^ mice. Our data reveal that mDia1 colocalizes with the MT network in T cells and that the induction of MT polarization, stabilization and plus-end clustering at the leading edge following LFA-1 engagement are severely impaired in migrating mDia1^-/-^ T cells. These abnormalities are associated with altered MT dynamics, defects in CXCL12-induced inactivation of glycogen synthase kinase (GSK) 3β, and reduction in the level and polarized accumulation of it substrate, adenomatous polyposis coli (APC). Together these findings identify regulation of the GSK3β-APC signaling axis as an important mechanism whereby mDia1 drives the MT cytoskeletal remodeling required for T cell adhesion and motility.

## Methods

### Mice and reagents

All mice were bred and maintained at the Ontario Cancer Institute Animal Facility and used at 6-12 weeks old in accordance to Institutional Animal Care and Use Committee of University of Toronto. This study (AUP #1826.13) was approved by Ontario Cancer Institute Animal Care Committee (ACC/OCI), certified by the Canadian Council on Animal Care (CCAC) and (Provincial) Ontario Ministry of Agriculture, Food and Rural Affairs.

The C57Bl/6 mDia1^-/-^ mice were created as previously described [14]. To generate mDia1^-/-^ OT-II mice, mDia1^-/-^ homozygotes were bred with ovalbumin-specific TCR transgene mice (OT-II) and progeny backcrossed. B6.SJL (CD45.1) congenic mice were from Jackson Laboratory (Bar Harbor, ME). All mice were bred and maintained at the Ontario Cancer Institute Animal Facility and used at 6-12 weeks old in accordance to Institutional Animal Care and Use Committee of University of Toronto. Reagents: cycloheximide, MG-132, ovalbumin, complete Freund’s adjuvant and GSK3β assay kits (Sigma Aldrich); antibodies against α-tubulin antibodies (AbD, Raleigh, NC),EB1(H-7H), APC(C-20), Rac1, RhoA, cdc42, Erk1,Erk2,Akt, β-actin and acetylated tubulin (Santa Cruz Biotechnology, CA),detyrosinated tubulin (Millipore, Bedford, MA),phospho-APC (S2054) and pericentrin (Abcam, Cambridge, MA) and GSK3β and phospho-GSK3β (Ser9), phospho-Erk1/Erk2, phospho-Akt (Cell Signaling Technology, Danvers, MA), and CD45.1, CD45.2, mouse LFA-1(M17/4), integrin alpha 4/CD49d and L-selectin (BD Biosciences, Palo Alto, CA); FITC, Cy3 or Cy5 conjugated secondary antibodies (Jackson Immune Research Labs, West Grove, PA); CXCL12, CCL21, IL-2, and ICAM-1-Fc (R&D Systems, Minneapolis, MN); pan-T cell isolation kits (MiltenyiBiotec); CD4^+^ T cell enrichment kit (Stem Cell Technologies, Auburn, CA); and CFSE (5,6-carboxyfluorescein diacetatesuccinimidyl ester) and CMTMR (chloromethylbenzoylamino-tetramethylrhodamine) CellTracker™ probes (Molecular Probes, Eugene, OR). 

### T cell purification, stimulation, transfection, and flow cytometry analysis

T cells were purified from spleens of 6-8 week-old mDia1^-/-^ mice or littermate controls with the EasySep CD4^+^ T cell enrichment kit. To generate effector T cells *ex vivo*, purified splenic T cells were activated with immobilized anti-CD3ε (5μg/mL) and anti-CD28 (2μg/mL) antibodies (2 days) followed by 6-day culture in IL-2 (10U/ml) medium. For T-cell transfection, cDNA encoding GSK3βS9A was subcloned into pDsRed-Express-C1 and transfected into effector T cells (2x10^6^) by electroporation (Amaxa Nucleofector) and transfected cells were sorted 24 hours later. For flow cytometry, cells were suspended in staining buffer (PBS, 1% BSA) and incubated (30 min, 4°C) with FITC, PE or Apc-conjugated antibodies. To assess levels of APC phosphorylation, T cells were maintained for 6 hrs in RPMI-1640 medium supplemented with 1% FCS, resuspended with HEPES buffer containing 5mM Mg^2+^/1mM EGTA and seeded (2×10^5^ cells/well) onto ICAM-1-Fc (3µg/ml) or poly-lysine-coated dishes at 37°C for 0-30 minutes. Cells were fixed, permeabilized, then incubated (1 hour) on ice with PE-conjugated anti-phospho-APC antibodies and analyzed on a FACS-Caliber TM (Becton Dickinson).

### 2D-migration time lapse video microscopy and immunofluorescence analysis

For live 2D imaging, *ex vivo*-derived effector T cells were seeded (2×10^5^ cells/well) onto ICAM-1-Fc (3µg/ml) or poly-lysine-coated dishes (ibidi Corp) and incubated at 37°C for 0-30 minutes in HEPES buffer containing 5mM Mg^2+^/1mM EGTA. Differential interference contrast (DIC) images were acquired (Zeiss AxioObserver) with a CoolSnap HQ CCD camera at 15' intervals. Cell migration was analyzed using MetaMorph 7.6 software (Molecular Devices) and Image ProPlus 6.0 (Media Cybernetics) to generate videos, plot cell tracks and determine the migratory parameters. The migration parameters were calculated from data on 30-50 cells in 4 independent experiments and using the mean value from all 4 experiments. Cells migrating more than 30μm in 20 min were scored to determine straightness and cell movement directionality. Parameters assessed were: velocity (cell centroid movement in μm/minute along total path length); displacement (linear distance between first and last measured positions); straightness index (net distance traveled divided by total linear distance traveled). 2D-migration tracks were generated by manually tracing the outline of each cell at 15 second intervals. Traces of T-cell boundaries were generated with images selected at 60 second interval from the time lapse videos, each successive time frame layered using Photoshop software. For immunofluorescence analysis of T cell polarization, migrating T cells were fixed with cold methanol, permeabilized (1% Triton X 100, 3 minutes), immunostained with FITC-phalloidin and Cy5-conjugated anti-α-tubulin plus Cy3-conjugated anti-pericentrin antibodies or mouse anti-mDia1 antibodies and/or DAPI and the confocal images analyzed with Leica TCS SP2 inverted microscope. To quantify T-cell polarization, average percentages of polarized cells in 10 microscopic fields (20-50 cells/field) were determined. Polarization was determined by two criteria: 1) ratio of x to y, where x is the longest distance across cells (from head to tail) and y is the greatest width perpendicular to x and 2) presence of diametric polarization of F-actin at the front and tail of the elongated cell.

### Two-photon microscopy

T cells (5x10^6^) were labeled for 10 minutes (RT) with 2 μM CFSE and 10 μM CMTMR and the mixed (1:1) CFSE- and CMTMR-labeled mDia1^-/-^ and WT T cells IV-injected into recipient C57Bl/6 mice. Mice were sacrificed 24 hours later and superficial cervical and axillary lymph nodes collected and placed in perfusion medium (RMPI-1640-phenol red)at 37°C. Lymph nodes were imaged on a 37°C-heated stage (Zeiss LSM 510 META NLO) and a Chameleon pulsed femtosecond laser excited at 810 nm, with emissions collected at 500-550 nm (CFSE) and 565-615 nm (CMTMR). For 3-D imaging, each xy plane spanned 256×256 μm (3 μm spacing, 60 μm depth, 20 xy planes/z-stack), z stacks imaged 40s apart for 15 minutes and the data then analyzed with Velocity software (Perkin Elmer). Manual cell tracking identified each cell centroid within images at successive times [18].

### T-cell adhesion and transmigration assays

For T-cell adhesion assay, purified mDia1^-/-^ and WT T cells were suspended in RPMI-1640/0.1% BSA and loaded on ICAM-1-Fc (0, 2.5, 5.0 μg/mL) or IgG (2.5μg/mL)-coated plates at 37°C for 1 hour in the presence of CXCL12 or CCL21 (100 nM) and centrifuged (200*g*, 10 min). After 30 min, cells were washed with warm RPMI-1640/0.1% BSA and detached with cold EDTA/PBS. Calibrite beads (5,000/ml) were added, and total cell number calculated by flow cytometry [19]. For transmigration assays, Purified T cells (2×10^6^) were loaded into trans-well chambers (Costar) coated with various concentrations of ICAM-1-Fc and placed in 24-well plates with various concentrations of CCL21 or CXCL12. After 4 hours at 37°C, lower chamber cells were collected and counted. 

### Homing of T cells to antigen-challenged skin

OVA-specific effector T cells were generated by incubating splenic T cells from mDia1^-/-^ or WT OT-II mice with OVA_323-339_ peptide loaded on irradiated splenocytes (T-cell depleted) in IL-2- supplemented medium for 6 days. The resultant OVA-specific effector T cells (CD45.2) were purified and IV-injected (5x10^6^) into B6.SJL mice (CD45.1) followed by intradermal antigen challenge in one ear with OVA-peptide (20μg in incomplete Freund’s adjuvant (IFA) versus PBS/IFA (control) in the other ear. The inflamed tissues were harvested 6 days later, digested with collagenase (100U/ml) and DNAse 1 (50U/ml), and accumulation of donor cells (CD45.2) in challenged ears assessed by immunostaining with fluorescently-labeled anti-CD4 and anti-CD45.2 antibodies and flow cytometric analysis. 

### Immunoprecipitation, immunoblotting and in vitro kinase assay

T cells were lysed and immunoprecipitated with primary antibody and immunoblotted sequentially with primary antibody and goat anti-rabbit horseradish peroxidase (Bio-Rad) as previously described [20]. To assess GSK3β kinase activity, T cells (5x10^6^) were stimulated with CXCL12 (100ng/ml) for indicated times, lysates prepared and immunoprecipitated with anti-GSK3β antibody, and the immunoprecipitated kinase was then incubated for different times with substrate solution. Samples were spotted onto cellulose membranes and radioactivity measured by Cerenkov mode. To assess RhoA/Rac1/cdc42 activation, cell lysates were incubated with GST-rhotekin-RBD or GST-PAK1-PBD fusion proteins (Santa Cruz) bound to glutathione sepharose beads and the precipitated GTP-Rho or Rac or cdc42 then detected by anti-RhoA, Rac1 or Cdc42 immunoblotting analysis. For assessing mDia1 association with GSK3β and LFA-1, T-lymphoblasts (2.5x10^6^) were plated on ICAM-1 (3 μg/ml)-coated dishes and stimulated with 100 ng/ml CXCL12 for 30 min. The cells were then suspended in 0.5 ml ice-cold lysis buffer (1% Triton X-100, 150 mM KCl, 150 mM NaCl, 1 mM PMSF and 1 μg/ml each of aprotinin, leupeptin and pepstatin). After 1 hr on ice, unlysed cells were removed by centrifugation at 12,000g for 30 min at 4°C and the lysates then precleared by incubation with Protein A Sephorase 6B beads (Amersham Pharmacia) for 1 hr at 4°C followed by an additional 2 hr incubation at 4°C with anti-mDia1 antibody or control IgG. The immune complexes were then collected by centrifugation, washed four times with lysis buffer and eluted by boiling in Laemmli sample buffer. For immunoblotting analyses, aliquots of total cell lysate or immunoprecipitated proteins boiled in Laemmli buffer were electrophoresed through 10% SDS-polyacrylamide and transferred to nitrocellulose (Schleicher & Schuell). After blocking with 3% BSA, filters were placed for 2 hr in cold TBST buffer (150 mM NaCl, 10 mM Tris-HCl, pH 7.4, 0.05% Tween 20) with 1% dry milk and sequentially incubated with anti-mDia1, anti-GSK3β or anti-LFA-1 antibodies and horseradish peroxidase-conjugated secondary antibody (Bio-Rad). Immunoreactive bands were visualized by ECL chemiluminescence. 

### Quantification of microtubule dynamics

Mouse embryonic fibroblasts (MEFs) were prepared from E13.5 day mDia1^-/-^ and wild-type littermate embryos and cultured in DMEM containing 10% fetal bovine serum (FBS). Cells (2×10^4^/well) were seeded onto ICAM-1-Fc (3µg/ml) or poly-lysine-coated dishes for 6-9 hours, transfected with EB1-GFP-expression vectors using Lipofectamine (Clonetech) and then cultured 24 more hours. Live cell imaging of mDia1^-/-^ and wild-type MEFs expressing EB1-GFP were then acquired using a Yokogawa spinning disc confocal microscope equipped with a PlanApo 60X oil objective (N.A. 1.42) (Quorum, Canada). Time-lapse images were acquired using a 491 nm diode laser and 0.3 seconds exposure times. Images were analysed and converted into movies using Velocity (Perkin Elmer, Version 6.0) software. Quantification of the number of microtubule nucleation events, tracking of microtubule movement trajectories, and computation of microtubule growth, and shortening rates and time spent in pausing were performed using the MOSAIC 2D/3D particle tracker [21]. *Bona fide* microtubules were selected by setting the selection algorithm to accept only local maxima of bright pixels in the upper 80^th^ percentile of all identified particles. Each EB1-GFP bright pixel was assigned a 3-pixel radius centroid from the brightest point and was quantified as one microtubule head. Analysis was conducted on movie lengths of 300 seconds that were captured at a rate of 2.98 frames/second. To exclude microtubules moving in and out of *z*-plane, events shorter than 25 frames were rejected from analysis. Microtubule growth/shortening rates were determined by using coordinate change between frames. Conversion from pixel speed to µm/min was calculated using a factor of 6.024 pixel/µm, with data points at speeds higher than 120 µm/min discarded. Movement was classified as either growth, shortening or pausing, based on movement, speed and direction. Change of direction was defined as a movement of more than 160-200 degrees from the current trajectory. Classifying using a k means clustering of changes in coordinates gave similar results to the direction-based classifier. 

### Statistical analysis

Data are expressed as the mean ± SEM unless otherwise specified. Unpaired two-tailed *t* tests were used for comparison of group means for continuous variables. Two-way analysis of variance (ANOVA) followed by the Bonferroni Dunn post-hoc test were used to assess differences in the mutant and wild-type cell responses at varying times after stimulation or stimulatory doses. Statistical calculations were performed using SPSS 16.0 software for Windows (SPSS Inc., Chicago, IL), with *p* values < 0.05 considered statistically significant. 

## Results

### mDia1 is required for T cell migratory polarization and *in vivo* trafficking

**Figure 1:**
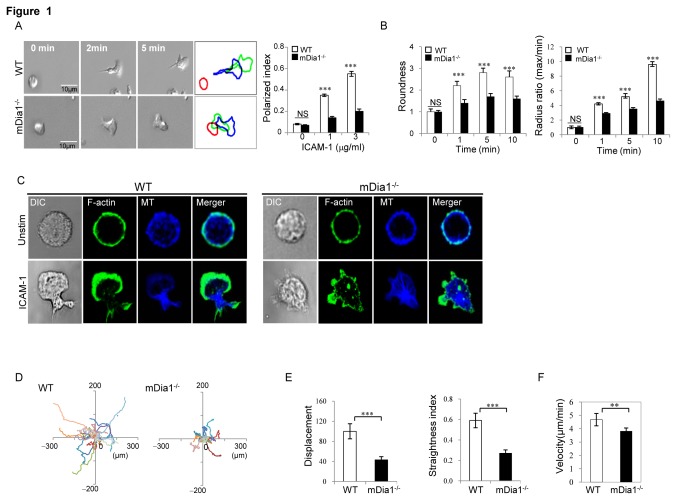
mDia1 is required for LFA-1-drivenT cell polarization and motility. In vitro differentiated mDia1^-/-^ and wild-type (WT) effector T cells were loaded on chamber slides coated with ICAM-1-Fc (3μg/ml) in the presence of Mg^2+^/EGTA (5mM/1mM) and cell movement tracked by 2D time-lapse video microscopy. (**A**) *Left*: Representative DIC images from videos of mDia1^-/-^ and wild-type migrating T cells. Outlines of the cells at 0 (red), 2 (blue) and 5 minutes (green) after loading reveal the cell migration paths. *Right*: The percentages of T cells morphologically polarized in response to ICAM-1 stimulation. Data are expressed as the mean values ± SEM and are representative of 3 independent experiments. ***, p<0.001 by two-way ANOVA. (**B**) *Left*: Cell roundness at varying times after stimulation, as determined by the following formula: (perimeter2)/(4* pi* area). *Right*: Cell radius ratios at varying times after ICAM-1 contact, as determined by maximal radius/minimal radius. Data expressed as mean values ± SEM and are representative of 3 independent experiments. ∗∗∗, *p*<0.001 by two-way ANOVA. (**C**) Representative confocal images of mDia1^-/-^ and wild-type cells migrating on ICAM-1-coated slides and immunostained with FITC-phalloidin or Cy5-conjugated with anti-α-tubulin antibody. (**D**) 2D migratory tracks over a 20 minute period are shown for individual control and mDia1^-/-^ T cells. (**E** & **F**) Migratory parameters are shown for mDia1-/- and wild-type cells, including, displacement: linear distance between first and last measured position; straightness index: the net distance traveled divided by total linear distance traveled; and velocity: centroid movement of the cell along the total path length. Values shown represent means ±SEM of triplicate evaluations and/or evaluation of 50-100 cells/condition and are representative of at least four independent experiments. **, p<0.01, ***, p<0.001.

To explore the influence of mDia1 on T cell polarization, we first assessed mDia1 effects on the migratory behaviour elicited in T cells in response to ICAM-1 engagement. Capitalizing on the availability of mDia1^-/-^ mice, time-lapse video microscopy was used to compare tracking plots generated by the movement of mDia1^-/-^and wild-type T-lymphoblasts on ICAM-1-coated substrate. Analyses of these images revealed markedly (~60%) less efficient polarization of the mutant relative to wild-type cells in response to ICAM-1, tracking of the mDia1^-/-^cell boundaries revealing their development of multiple unstable pseudopods, lack of the elongated morphology, distinct leading edges and uropods apparent in wild-type cells (Figure 1A), and concomitant reductions in roundness and radius ratio values (Figure 1B). Immunostaining of the mDia1^-/-^ cells also revealed their formation of multiple, irregular pseudopods in response to ICAM-1 contact, F-actin and tubulin appearing randomly distributed in these cells in contrast with the organized accumulation of F-actin at the leading edge and tubulin at the cell posterior observed in wild-type cells (Figure 1C). Consistent with these findings, while control T cells exhibited robust and sustained motility on ICAM-1, displacement as well as straightness indices were dramatically reduced in the mutant T cells (Figure 1D & Videos S1 and S2), although velocity was only modestly diminished compared to wild-type T cells (Figure 1E). 

**Figure 2:**
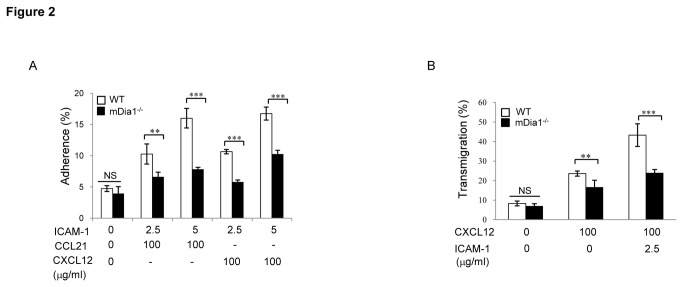
mDia1 is required for LFA-1-mediated adhesion and transmigration. (**A**) CD4^+^ T cells purified from 6-8 weeks old mDia1^-/-^ mice and wild-type littermates were plated on indicated concentrations of ICAM-1-Fc or IgG-coated 96-well-plates in the presence of 100 nM CCL21 or CXCL12. Percentages of adherent T cells were evaluated before and 30 min after stimulation by fluorescent-bead-based flow cytometric cell counting. (**B**) Migration of mDia1^-/-^ and wild-type CD4^+^ T cells towards wells containing 100nM CXCL12 was evaluated as above using transwells containing ICAM-1-coated filters. Migration was measured as the percentage of total cells loaded and collected from the chemoattractant-containing wells. Data for A and B are expressed as the mean values ± SEM and are representative of 4 independent experiments. ***, p<0.001, **, p<0.01 by two-way ANOVA. NS: not significant.

As these data suggest essential roles for mDia1 in the modulation of cytoskeletal dynamics coupling LFA-1 engagement to altered T cell polarization, effects of mDia1 deficiency on LFA-1-mediated T cell adhesion and transmigration were also examined. Although levels of adhesion molecules and chemokine receptors were normal on mDia1^-/-^T cells (Figure S1), these cells showed markedly reduced adherence to ICAM-1 substrate in the context of stimulation with either of two chemokines CCL21 or CXCL12 (Figure 2A). Migration towards CCL21 or CXCL12 across ICAM-1-coated inserts in transwell assays (Figure 2B) was also dramatically reduced in mDia1^-/-^compared to wild-type cells. Thus mDia1 appears to play essential roles in modulating both the polarization and adhesion/motility evoked in T cells by LFA-1-ICAM-1 engagement. 

**Figure 3:**
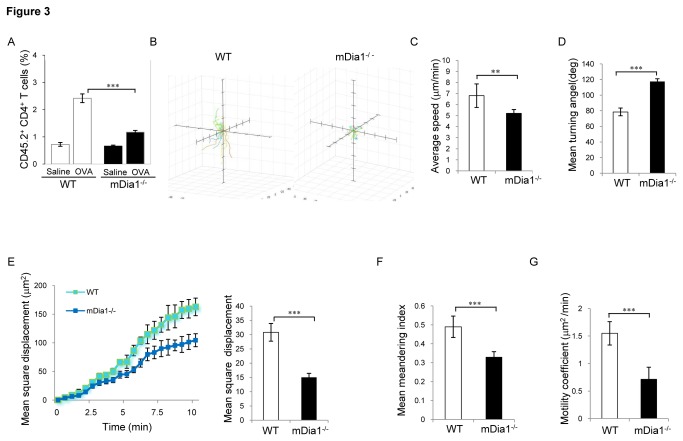
*In vivo* migratory responses are impaired in mDia1-deficient mice. (**A**) Effector T cells were generated by *ex* *vivo* stimulation of Ova-specific T cells from mDia1^-/-^ OT-II or OT-II TCR mice (CD45.2^+^) with Ova peptide-loaded splenocytes for 6 days and the effector cells then injected into B6.SJL recipient mice (CD45.1) followed by intradermal challenge with OVA-peptide in incomplete Freund’s adjuvant. The accumulation of donor CD45.2 mDia1^-/-^ and wild-type effector T cells in Ova antigen-challenged skin was determined by immunostaining of single skin cell suspensions with PE-conjugated-anti-CD45.2 and Apc-conjugated-anti-CD4 antibodies. (B-G) Intranodal T cells migration. Naive CD4^+^ T cells purified from the spleens of mDia1^-/-^ and wild-type mice were labeled with CFSE or CMTMR and the mixture (1:1) of CFSE-labeled mDia1^-/-^ and CMTMR-labeled wild-type T cells intravenously injected into C57BL6 mice. Lymph nodes from recipient mice were imaged 24 hrs later by time-lapse two-photon confocal microscopy. The migratory parameters of migrating T cells in time were calculated from the movies using Velocity software. (**B**) Representative three-dimensional tracks of mDia1^-/-^ and wild-type T cells over a 20 min period. Each colored line represents a single T cell track. (**C**) Relative frequency of average velocity calculated from the net distance traveled during each time interval (displacement/time during a single time step) of a cell over 20 min; (**D**) Frequency of mean turning angles from which a cell deviates between successive time steps over 20 min; (**E**) Frequency of mean square displacement (left panel),a measure of the average distance a given particle in a system travels, and average mean square displacement over 20 min (right panel) are shown for wild-type and mDia1^-/-^ cells. (**F**) Mean meandering index, a measurement of directionality, was calculated by dividing the distance a cell traveled by the track length. (**G**) Motility coefficient (M) of mDia1^-/-^ and control T cells. M is a measure for how fast cells displace from their starting positions during a random walk process, analogous to the diffusion coefficient for Brownian motion (M=displacement^2^/6*t*). Data are expressed as the mean values ±SEM and are representative of at least 4 independent experiments. ∗∗ indicates *p*<0.01, *** indicates *p<0.001*.

To ascertain whether the defects in polarization of mDia1^-/-^T cells translate into altered migratory properties *in vivo*, mDia1 influence on trafficking of T cells during antigen-evoked inflammatory responses was also explored. For this analysis, T cells from mDia1^-/-^and wild-type (CD45.2^+^) mice expressing the OT-II TCR were stimulated *ex vivo* with OVA_323-339_-pulsed splenocytes and adoptively transferred into CD45.1^+^ mice, and their migration into OVA-challenged tissue evaluated. Results of this analysis revealed a 2-fold reduction in accumulation of the mDia1^-/-^ compared to wild-type-derived donor CD4^+^ T cells at OVA-challenged inflammatory sites (Figure 3A). We further examined the effect of mDia1 on T cell intranodal motility by adoptively transferring a mixture of CFSE-labeled mDia1^-/-^ T cells and CMTMR-labeled WT cells into recipient mice and examining movement of these cells within lymph nodes using two-photon laser microscopy (Video S3). Compared to control cells, the mDia1^-/-^ T cells showed markedly shorter track lengths as well as lower migratory speeds (median velocity: 5.20 ± 0.35 μm/min in mutant versus 6.80 ± 1.06 μm/min in wild-type cells) (Figure 3B & C). The mDia1^-/-^ T cells also exhibited higher frequency of directional change and a 50% reduction in mean square displacement (Figure 3D & E). Consistent with these findings, both the mean meandering index, a measure of direct distance : total distance ratio that indicates the extent to which cells consistently move forward in the same direction and the average motility coefficient, a measure of the volume a cell surveys over time [22], were significantly reduced in mDia1^-/-^ T cells as compared with wild-type T cells (Figure 3F & G), suggesting a role for mDia1 in enabling both motility and directional persistence during T cell intranodal migration. Thus mDia1 appears to be required for the T cell migratory polarization evoked by LFA-1-ICAM-1 interaction and, in its absence, LFA-1-mediated T cell adhesion/transmigration and *in vivo* trafficking are profoundly impaired.

### mDia1 is required for LFA-1-induced MT polarization and stabilization in T cells

**Figure 4:**
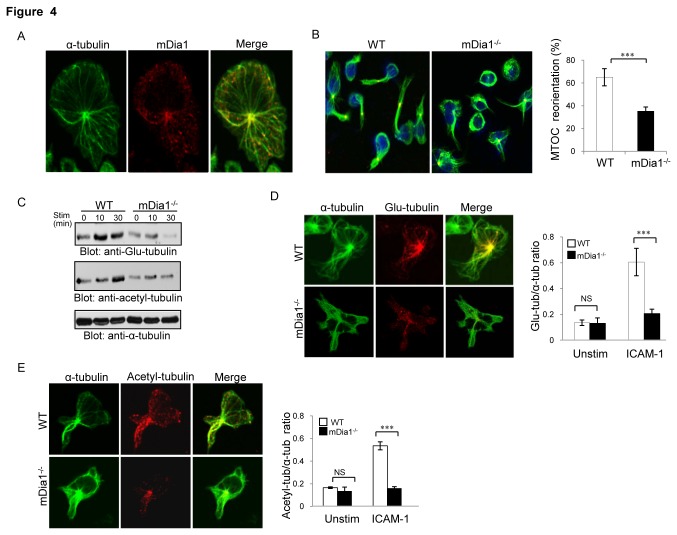
mDia1 regulates LFA-1-mediated T cell MT polarization and MT stabilization. *Ex-vivo* derived wild-type or mDia1^-/-^T lymphoblasts were stimulated with ICAM-1 plus Mg^2+^/EGTA for 30 min followed by staining with fluorescently-labeled antibodies. (**A**) Representative immunofluorescence images showing the intracellular localization of mDia1 in ICAM-1-stimulated wild-type T cells stained with Cy3-labeled anti-mDia1 antibody and FITC- or Cy5-conjugated anti-α-tubulin antibody. (**B**) *Left*: MTOC reorientation in mDia1^-/-^ and wild-type T cells stained with FITC-conjugated anti-α-tubulin and Cy3-conjugated anti-pericentrin antibodies. DAPI (blue) staining was used to show the nucleus. *Right*: MTOC reorientation was quantitated by determining the number of cells among 100 cells scored showing either no or aberrant MTOC reorientation. (**C**) WT or mDia1^-/-^ T cells (5x10^6^) were stimulated with ICAM-1/Mg2^+^ (3μg/ml) or control IgG for the indicated times, lysed and the lysates then subjected to immunoblotting analysis with anti-Glu-tubulin or anti-acetyl-tubulin antibodies and reprobed with anti-α-tubulin antibody as loading control. (**D** & **E**) *Left*: WT or mDia1^-/-^ T cells migrating on ICAM-1-coated plates were stained with anti-α-tubulin antibody and anti-Glu-tubulin antibody (**D**) or anti-acetyl-tubulin (**E**) antibody. *Right*: Calculation of the ratios of α-tubulin versus Glu-tubulin or acetyl-tubulin fluorescence intensities (analyzed using Image-Pro Plus 6.0 software). For all experiments, values shown represent means ±SEM and are representative of at least 4 independent experiments. ∗∗∗, *p<0*.*001*.

Given the demonstration of mDia1 involvement in cellular polarization and motility, we next explored the potential for mDia1 to modulate MT rearrangements evoked by LFA-1-ICAM-1 interaction. An initial examination of mDia1 distribution in relation to the MT cytoskeleton of T cells migrating over ICAM-1 revealed that mDia1 was localized along the radical MT array and concentrated at the distal ends of MTs and within the MTOC (Figure 4A). Dramatic polarization of the MT cytoskeleton was also evident in these cells, the MTs organized along the directional axis and the pericentrin-containing MTOC retracted posteriorly towards the uropod in ^~^60% of wild-type T cells (Figure 4B). By contrast, posterior retraction of the MT cytoskeleton was severely impaired in mDia1^-/-^ cells, with the MTOC distributing randomly in the majority of these cells, indicating mDia1 in regulates MT asymmetry during LFA-1-mediated T cell polarization.

The cellular polarization and MT asymmetry associated with migratory responses have been shown to require not only spatially focused redistribution of the MT cytoskeleton, but also the generation of stabilized MTs aligned towards the cell leading edge [23]. MT stabilization is associated with a number of tubulin post-translational modifications, including detyrosination and acetylation [24]. Thus, to assess whether mDia1 influence on integrin-evoked T cell polarization relates to effects on formation of stabilized MTs, levels of detyrosinated tubulin (Glu-MTs) and acetylated tubulin (acetyl-MTs), two indicators of stabilized MTs, were compared in the mutant and wild-type cells by immunoblotting analysis. This analysis revealed levels of Glu- and acetyl-MTs to be markedly increased in wild-type T cells following ICAM-1 exposure, but to be only marginally increased in similarly-treated mDia1^-/-^ cells (Figure 4C). Consistently, Glu-and acetyl-MT accumulation in ICAM-1-stimulated mDia1^-/-^ T cells were markedly reduced as compared to wild-type T cells (Figure 4D & E). Thus mDia1 appears to play a key role in the MT remodeling and stabilization elicited by T cell interaction with ICAM-1.

### MT plus-end stabilization and accumulation at the leading edge are impaired in mDia1^-/-^ T cells

**Figure 5:**
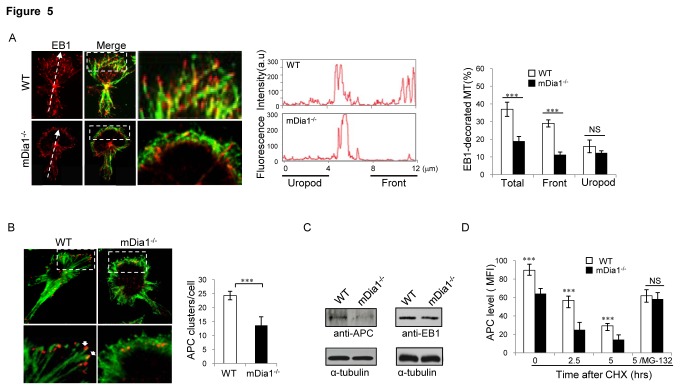
mDia1 is required for induction of MT plus-ends dynamics downstream of LFA-1-ICAM-1 interaction. WT or mDia1^-/-^ T lymphoblasts were loaded onto ICAM-1(3µg/ml)-coated plates for 30 min, fixed and immunostained using FITC-conjugated anti-α-tubulin and Cy3-conjugated anti-EB1 antibody. (**A**) *Left*: Representative images of WT and mDia1^-/-^ T cells migrating on ICAM-1 show EB1 clustering with MTs plus ends in polarized T cells. The images shown in the far right panel are enlarged versions of the boxed areas shown in the middle panels. *Middle* *graph*: Quantitation of the fluorescence intensities of EB1 staining across the cell axis from uropod to the leading edge of polarized T cells (depicted by dashed arrow in far left image).*Right* *bar* *graph*: Quantitation of the number of cells among 100 cells assessed showing EB1 binding to the tips of growing MT plus-ends at the leading edge cell periphery. (**B**) *Left*: Representative images showing the distribution of APC in wild-type and mDia1^-/-^ T cells migrating on ICAM-1. *Right*: Quantitation of the number of cells among 100 cells counted showing APC clustering at the leading edge. (**C**) Lysates prepared from mDia1^-/-^and WT T cells were subjected to SDS-PAGE and immunoblotted with anti-APC or anti-EB1 antibodies followed by anti-α-tubulin antibody. (**D**) Wild-type or mDia1^-/-^ T lymphoblasts were treated for the indicated times with 10μM cyclohexamide in the absence or presence of the proteosome inhibitor MG-132 (10μM). The cells were then fixed and permeabilized for immunostaining with anti-APC antibody followed by flow cytometric analysis. Data are expressed as the mean fluorescence intensity (MFI). Values represent mean ± SEM and all data for A-D are representative of 4 independent experiments. ∗∗∗, *p*<0.001 by two-way ANOVA, NS: not significant.

While the acquisition of polarized cell morphology is thought to require stabilized MTs, the MT behaviours underpinning polarized migration are now recognized as complex and shown in some cell systems to also depend on localized MT dynamic instability [25,26]. To evaluate the extent to which MT dynamics are effected by mDia1 in polarizing cells, we evaluated MT growth events in mDia1^-/-^ and wild-type mouse embryonic fibroblasts (MEFs) using expression of GFP-EB1 to track plus-ends of elongating MTs [26]. Confocal imaging of these cells (Videos S4 and S5) revealed average growth rate of MTs to be comparable in wild-type (29.63±6.04μm/min) and mutant (31.39±7.95μm/min) cells (Figure S2A). By contrast, average shortening rates for MTs were higher in the mutant (37.19±11.26μm/min) than wild-type (30.97±7.97μm/min) cells and the mutant MTs spent higher percentages of time in shortening and relatively less in pause, suggesting increased dynamic activity of the mDia1^-/-^ MTs (Figure S2B & C). Consistent with this possibility, immunofluorescence analysis of anti-EB1-stained wild-type T cells migrating over ICAM-1, revealed a preponderance of EB1-decorated MTs at the leading edge, localized in a polarized fashion in parallel tracks with their distal ends pointed perpendicular to the cell periphery (Figure 5A). By contrast, EB1-decorated MT plus ends were significantly reduced in mDia1^-/-^ T cells, a quantitative scan of the EB1 fluorescence signals showing EB1 staining in these cells to be reduced, most prominently at the leading edge where the MT plus-end arrays appeared disorganized and unable to extend into peripheral cell protrusions (Figure 5A). These data suggest integral roles for mDia1 in modulating MT dynamics as is consistent with a critical role for this formin in MT stabilization.

Because APC has been shown to promote MT plus-end stabilization in fibroblasts [27,28], we also explored the effects of mDia1 on APC redistribution following T cell contact with ICAM-1. This analysis revealed that APC accumulated as punctuate clusters at MT plus-ends in close proximity to membrane protrusion in polarized wild-type T cells migrating on ICAM-1 (Figure 5B). By contrast, the formation of APC clusters in mDia1^-/-^ cells was reduced by at least 50% compared to wild-type T cells. To explore the basis for mDia1 effects on APC MT clustering, we examined the EB1 and APC levels in mDia1^-/-^ T cells by immunoblotting analysis. Results of this analysis revealed levels of APC protein to be markedly reduced in the mDia1^-/-^ T cells, while EB1 protein levels were essentially comparable in mutant and wild-type cells (Figure 5C). To ascertain whether this defect reflects decreased APC expression or altered rates of APC turnover, levels of EB1 and APC transcripts were assessed using quantitative PCR and found to be comparable in wild-type and mDia1^-/-^ T cells (data not shown). However, comparison of the APC turnover rates in the mutant and wild-type T cells by assessing APC protein levels after cycloheximide treatment, revealed a marked increase in APC degradation in mDia1^-/-^ compared to wild-type T cells (t½ =1.98 hr versus 2.46 hr), with APC levels restored to normal in mutant cells by treatment with the MG132 proteosome inhibitor (Figure 5D). Together these findings reveal that mDia1 is required for MT plus-end stabilization and for APC stability and plus-end accumulation at the leading edge of migrating T cells and suggest that the impaired MT polarization and stabilization observed in mDia1^-/-^ T cells reflect, at least in part, abnormalities in MT plus-end dynamics.

### Loss of mDia1 impedes induction of GSK3β inactivation and APC phosphorylation in migrating T cells

**Figure 6:**
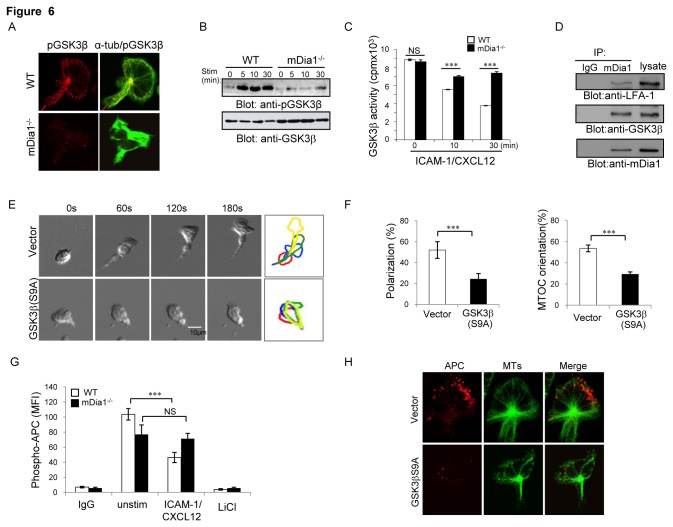
Inducible inactivation of GSK3β is disrupted in migrating mDia1^-/-^T cells. (**A**) Immunofluoresence analysis of wild-type and mDia1^-/-^ T-lymphoblasts loaded on ICAM-1/CXCL12-coated plates for 30 min and stained with Cy3-labeled anti-phospho-GSK3β antibody (red) and FITC-conjugated anti-α-tubulin antibody. (**B** & **C**) mDia1^-/-^ and WT T cells were stimulated with ICAM-1/CXCL12 for the indicated times and the lysates then either (**B**) subjected to SDS-PAGE followed by sequential immunoblotting analysis with anti-phospho-GSK3β (S9A) and anti-GSK3β antibodies (**C**) or subjected to immunoprecipitation with anti-GSK3β antibody or control IgG and the precipitated samples then incubated with [γ-^32^P]-ATP and assayed for incorporated radioactivity. Values represent means ± SEM, ***, p<0.001 by two-way ANOVA. (**D**) Lysates prepared from T-lymphoblasts (2.5x10^-6^) were immunoprecipitated with anti-mDia1 antibody or control IgG and the lysates as well as immunoprecipitated proteins then resolved on SDS-PAGE and subjected to sequential immunoblotting with anti-mDia1, anti-GSK3β or anti-LFA-1 antibodies. (**E**) Representative differential interference contrast images of pEGF-GSK3β or pEGF-expressing T cells migrating on ICAM-1/Mg^2+^-coated substrate. WT T-lymphoblasts were transfected with either pEGFP-GSK3β (S9A) or pEGFP vector for 24 hours and the GFP^+^-cells then isolated by cell sorting, loaded over ICAM-1-coated plates and their movement tracked using time-lapse microscopy. Outlines of the cells at 0 (red), 1 (blue), 2 (green) and 3 minutes (yellow) post plating indicate the cell migration paths. Scale Bars: 10μm. (**F**) Bar graphs showing the percentages of morphologically polarized (left) and MTOC reorientated (right) EGF-GSK3β-(S9A) or EGF-expressing T cells. Values represent means ± SEM and all data are representative of at least 4 independent experiments. ∗∗∗, *p*<0.001. (**G**) WT and mDia1^-/-^ T-lymphoblasts (5x10^6^) were untreated or treated with ICAM-1/CXCL12 or the GSK-3 inhibitor LiCl (20mM) and APC phosphorylation assessed by evaluating mean fluorescence intensity (MFI) of anti-phospho-APC antibody staining. Values represent means ± SEM, ***, *p*<0.001, NS, not significant. (H) Immunofluoresence analysis of wild-type T lymphoblasts transfected with either GSK3β (S9A) or control vector and loaded on ICAM-1/CXCL12-coated plates for 30 min and stained with Cy3-labeled anti-phospho-GSK3β antibody (red) and Cy5-conjugated anti-α-tubulin antibody. All data are representative of at least four independent experiments.

The glycogen synthesis kinase (GSK) 3β has been shown to phosphorylate MT plus-end binding proteins so as to regulate their plus-end targeting and thereby modulate MT dynamics [29-32]. Thus the potential relevance of GSK3β to defective MT polarization in mDia1^-/-^ T cells was also explored. GSK3β generally exists in a non-phosphorylated and active state in resting cells and is inactivated by phosphorylation on Ser9 following cell stimulation [33]. Immunofluorescence analysis showed that in Ser9 phosphorylated (i.e. inactive) GSK3β (pGSK3β) accumulated at the MTOC, leading edge and uropod in wild-type T cells migrating on ICAM-1/CXCL12-coated substrate (Figure 6A). By contrast, pGSK3β level was significantly reduced in migrating mDia1^-/-^ cells, suggesting an impairment in the induction of GSK3β phosphorylation in these cells. Consistent with this possibility, immunoblotting analysis revealed that ICAM-1/CXCL12 stimulation induced rapid GSK3β phosphorylation and decrease in GSK3β kinase activity in wild-type T cells, but the inducible increase in pGSK3β level and concomitant decrease in GSK3β activity were diminished in mDia1^-/-^ T cells (Figures 6B and C). By contrast, induction of Akt, Erk1/2, and the Rac, cdc42 and Rho GTPases appeared comparable in WT and mDia1^-/-^ T cells (Figure S3), suggesting that mDia1 effects on GSK3β do not reflect upstream modulation of these signaling events. These data indicate that mDia1 is required for GSK3β phosphorylation and inactivation in T cells responding to promigratory stimuli. In keeping with this conclusion, mDia1 was also coimmunoprecipitated with GSK3β and LFA-1 from wild-type T-lymphoblasts (Figure 6D), indicating that mDia1 associates with both the kinase and LFA-1 and, as such, is well positioned to influence the effects of LFA-1-engagement on GSK3β activity.

To further explore the biologic significance of mDia1-mediated GSK3β regulation, the relevance of GSK3β activity to T cell polarization and motility was examined using cells ectopically-expressing constitutively active GSK3β (GSK3βS9A). As revealed by time-lapse video microscopic images (Figure 6E & F), GSK3βS9A expression was associated with impaired T cell polarization and migration, the GSK3βS9A transfected cells moving slower (V_mean_ = 2.82 ± 1.06) than control cells (V_mean_ = 4.38 ± 1.10) and showing shorter migration tracks (data not shown) and reduced MTOC reorientation. Thus GSK3β inactivation appears critical to T cell migration as is consistent with mDia1 capacity to both downregulate this kinase activity and promote the MT dynamics needed for migratory polarity in T cells. 

Because GSK3β-mediated phosphorylation of APC plays an essential role in regulating its stability and localization [28], we further explored the relevance of mDia1 in mediating GSK3β-dependent APC phosphorylationin T cells. We first examined the effect of GSK3β inhibitor on APC phosphorylation in T cells and found that the GSK3β inhibitor lithium chloride completely abolished APC phosphorylation in both wild-type and mDia1^-/-^ T cells (Figure 6G), indicating that GSK3β is required for induction of APC phosphorylation in T cells. We then assessed the effect of mDia1 deficiency on APC phosphorylation, an analysis that revealed the Ser/Thr phosphorylation of APC to be decreased by ^~^50% in wild-type T cells in response to promigratory cues (ICAM-1/CXCL12 stimulation), but to remain unchanged from the level observed in the unstimulated state in similarly-treated mDia1^-/-^ T cells (Figure 6G). Consistently, the formation of APC clusters was dramatically reduced in GSK3βS9A-expressing T cells transfectants compared with vector-transfected control T cells (Figure 6H). Together, these data imply that the impairment in activation-induced phosphorylation and inactivation of GSK3β in mDia1^-/-^ T cells, results in enhanced APC phosphorylation and degradation that in turn contributes to the disruption of MT positioning and dynamics in these cells. 

## Discussion

The microtubule cytoskeleton is integrally involved in coupling external stimuli to polarity-dependent cell processes [2,5]. However, the molecular mechanisms whereby MT rearrangements drive specific effector functions are not well defined in T cells. In this study, the role for mDia1 as a potential driver of the polarization and microtubule behaviours required for T cell migratory polarity was explored in mDia1-deficient T cells. Our data reveal lack of mDia1 to be associated with marked impairment in T cell adhesion, transmigration and in vivo trafficking. The mDia1^-/-^ T cells also manifest aberrancies in two-dimensional migration on ICAM-1, these cells showing erratic migratory patterns, and marked decreases in directional persistence of migration and displacement. These abnormalities are associated with profound impairment in the acquisition of polarized morphology, the mutant T cells exhibiting multiple, unstable pseudopod formation and failure to develop distinct anterior-posterior axis. Consistent with these observations and a prior report implicating mDia1 in centrosome orientation downstream of TCR engagement [16], the LFA-1-evoked MTOC reorientation and plus-end stabilization observed in migrating wild-type T cells were also severely reduced in mDia1^-/-^ cells. Thus mDia1 plays a central role in modulating the MT rearrangements associated with induction of T cell migratory polarization downstream of LFA-1-ICAM-1 engagement. 

The morphologic polarization underpinning cell migration is highly dependent on the dynamic reorganization of MT plus-ends. Although mDia1 has been shown to bind directly with MTs [34], its roles in regulating MT reorganization are not fully understood. By tracking EB1-GFP-labeled MT plus-ends using time-lapse confocal microscopy, we found that rates of MT shortening and time spent shortening were markedly increased in mDia1^-/-^ compared to wild-type T cells, while the percentage of time spent in pause was relatively reduced in the mutant cells and their growth rates essentially comparable to those of wild-type cells. These findings reveal mDia1 deficiency to be associated with increased MT dynamic instability in migrating T cells. This result is consistent with the reduced numbers of stabilized and EB1-decorated MTs detected at the leading edge of mDia1^-/-^ T cells and identifies a critical role for mDia1 in regulating the MT plus-end dynamics and stabilization associated with T cell migratory polarization.

The mechanisms underlying MT plus-end stabilization are not fully understood, but involve molecular interactions between plus-end-associated proteins that enable MTs growing at the cell periphery to connect to cortical targets [25,35]. APC, a protein that plays key roles in MT attachment to the cell cortex, has been shown to promote MT stabilization at the leading edge of migrating fibroblasts by complexing with the mDia2 formin [36]. APC also cooperates with other plus-end-binding proteins to enhance plus-end capture by membrane-associated complexes [37,38]. Our findings in this study are consistent with these properties of APC, revealing the reduction of APC-MT clustering at the leading edge of migrating mDia1^-/-^ T cells to be associated with significant impairment in MT plus-end stabilization. Together with our finding that APC as well as EB1 associate with mDia1 in wild-type T cells (data not shown), the current data suggest that APC association with mDia1 is key to its participation in the MT plus-end growth and stabilization evoked at the anterior of T cells migrating over ICAM-1. This conclusion is supported by the colocalization of mDia1 with the MT arrays generated at the leading edge of polarizing T cells and also by a previous report identifying the formation of mDia1/EB1/APC complexes in migrating fibroblasts [36].

The localization of APC at MT plus-ends has been shown to depend on its phosphorylation status [28]. While the functions of GSK3β in T cells are not well defined, this kinase has been shown to play essential roles in regulating MT plus-end growth and stabilization in some cell types via modulation of MT plus-end-binding protein interactions. Our data show that in T cells, GSK3β undergoes phosphorylation and inactivation in response to promigratory signals and that in the absence of mDia1, GSK3β activity is increased. Considering the central roles for GSK3β in regulating MT interactions with plus-end binding proteins [28-30], the reduction in inducible downregulation of GSK3β activity and concomitant upregulation of APC phosphorylation and degradation observed in mutant T cells, identify attenuation of GSK3β inhibitory effects on APC as a mechanism whereby mDia1 can influence MT dynamics and stabilization. Consistently, inhibition of GSK3β abrogates APC phosphorylation, while expression of constitutively active GSK3β (S9A) disrupts MT polarization and formation of APC clusters at MT plus-ends in T cells migrating over ICAM-1. While this latter finding may reflect a deleterious effect of activating GSK3β in an inappropriate temporal context (i.e. constitutively), our data concur with previous reports showing that GSK3β phosphorylates selected MT plus-end-binding proteins and promotes their MT interactions and subsequent proteosomal degradation [29,30], and also with data showing GSK3β inactivation to be required for APC clustering with MTs [32]. Because APC stability and protein levels are reduced in mDia1^-/-^ relative to wild-type T cells, it is not possible to discern whether the absence of APC clusters at the MT plus-ends in the mutant cells is caused by APC hyperphosphorylation or by its reduced protein level. Nonetheless, results from this study support a critical role for mDia1 in regulating MT dynamics and suggest this role to be subserved, at least in part, by modulation of GSK3β-dependent APC MT plus-end clustering and stabilization in migrating T cells (Figure S4). 

The β2-integrin LFA-1 plays critical roles in mediating T-cell interaction with antigen-presenting cells and facilitates T-cell adhesion to the endothelium, a process that is important for lymphocyte extravasation and the homing to peripheral lymphoid and inflammatory tissues that supports antigen peptide-MHC recognition and cytotoxic killing [39,40]. The findings reported here identify mDia1 as an important modulator of LFA-1-mediated T cell responses, revealing LFA-1-mediated T-cell adhesion, transmigration and recruitment to antigen-challenged tissues to be impaired in mDia1^-/-^ mice and suggesting that defects in T cell homing observed in mDia1^-/-^ mice reflect, at least in part, mDia1 involvement in these LFA-1-mediated responses. While T cell movement within interstitial tissues such as the lymph node parenchyma is highly dependent on soluble chemokine gradient-derived guidance cues [41], β2-integrin activation has also been implicated in T cell interstitial motility, possibly by effects on adhesion/deadhesion that may confine the speed and directionality of migrating cells [42-44]. Because mDia1 has been previously implicated in both antigen and chemokine-evoked T cell responses [14-16], this formin is well positioned to participate in the integration of stimulatory signals evoked by antigen or chemokine receptors with those elicited by integrin receptor engagement. This possibility is consistent with the defects in T cell intranodal migration and trafficking in mDia1^-/-^mice, abnormalities that are likely to reflect combined effects of mDia1 deficiency on chemokine and integrin-mediated signaling. While the molecular pathways whereby mDia1 impacts on *in vivo* T cell trafficking require further dissection, the current data reveal mDia1 deficiency to be associated with polarization/migratory defects likely key to the attenuated T cell-dependent immune responses observed in mDia1^-/-^ mice [14,15] and suggest that mDia1 effects on the T cell MT cytoskeleton translate into a significant role for this formin in promoting the migratory polarization and likely other polarity-driven T cell behaviours required for effective immune responses.

## Supporting Information

Figure S1 **Expression of surface adhesion molecules and chemokine receptors in WT and mDia1^-/-^ T cells.** Histograms showing the expression of cell surface adhesion molecules and chemokine receptors on control and mDia1^-/-^ T cells. Lymph node cells from mDia1^-/-^ and wild-type (WT) mice were stained with the indicated fluorescently-labeled antibodies and analyzed by flow cytometry.
(TIF)

Figure S2 **Effect of mDia1 deficiency on microtubule dynamics.** (**A**&**B**) Graphs showing the growth (**A**) and shortening (**B**) rates of EB1-GFP-labeled MTs from wild-type or mDia1^-/-^ cells. (**C**) The percentages of pausing, shortening and growing times displayed by MTs at the cell cortex of mDia^-/-^ and WT cells. Data are presented as means ± SD (***, p<0.001). Data for A-C are representative of 3 independent experiments and are derived from analyzing about 300 MTs in 20 cells over a 5 min period.
(TIF)

Figure S3 **Analysis of mDia1 effects on proximal T cell signaling effectors.** (**A**&**B**) mDia1^-/-^ and WT T cells were left unstimulated or stimulated with CXCL12/ICAM-1 for 5 min and (**A**) the lysates then subjected to SDS-PAGE followed by immunoblotting with anti-phospho-PLCγ and anti-PLCγ antibodies, anti-phospho-Akt and then anti-Akt antibodies and anti-phospho-Erk1/Erk2 and then anti-Erk1/Erk2 antibodies; or (**B**) the lysates incubated with GST-rhotekin Rho-binding domain (to detect active Rho A) or GST-Pak1 protein-binding domain (to detect cdc42 or Rac1) fusion proteins immobilized on glutathione agarose beads and the precipitated proteins or whole cell lysates subjected to SDS-PAGE followed by immunoblotting with anti-Rac, cdc42 or RhoA antibodies.
(TIF)

Figure S4 **Schematic showing the proposed molecular pathway whereby mDia1 links LFA-1-engagement to MT stabilization and T cell polarization.** Data from this study reveal involvement in linking LFA-1-ICAM-1 engagement in T cells to induction of GSK3β Ser/Thr phosphorylation and consequent inactivation. Because activated GSK3β normally evokes APC phosphorylation and degradation, mDia1-mediated GSK3β inactivation enables APC to accumulate at the MT plus-ends and thereby facilitate MT stabilization and polarization. By this means, mDia1 promotes LFA-1-mediated adhesion and T-cell transmigration and may enable LFA-1 to cooperate with chemokine-dependent directional cues to facilitate interstitial T cell migration. The mechanism whereby mDia1 modulates GSK3β phosphorylation is unknown, but appears to operate downstream or independently of Akt. pAPC: phosphorylated adenomatous polyposis coli; GPCR: G-protein-coupled receptor; pGSK3β: phosphorylated glycogen synthase kinase B.
(TIF)

Video S1 **Time-lapse video showing migration of wild-type T lymphoblasts on an ICAM-1-coated plate in the presence of Mg^2+^/EGTA (1 sec video = 2.5 min real time).** The video is representative of five independent experiments.
(AVI)

Video S2 **Time-lapse video showing migration of mDia1-deficient T lymphoblasts on ICAM-1 in the presence of Mg^2+^/EGTA (1 sec video = 2.5 min real time).** The video is representative of five independent experiments.
(AVI)

Video S3 **Representative video showing the *invivo* interstitial migration of wild-type (green) and mDia1^-/-^ (red) T cells.** Naive T cells from mDia1^-/-^ and wild-type mice were labeled with CFSE and CMTMR, respectively and injected 1:1 intravenously into B6 mice. Mice were sacrificed 24 hours after injection and their cervical or axillary lymph nodes imaged with Zeiss LSM 510 META NLO using the FLUAR 20×/0.75 NA objective lens and Zeiss software for image acquisition. For 3-D time lapse imaging, each xy plane spanned 256×256 um at 3um spacing and 60um depth (20 xy planes in each z-stack). Each z stack was imaged at 20 second intervals over a period of 5 minutes. The data are representative of six independent experiments.
(MPEG)

Video S4 **Time-lapse video showing movement of EB1-GFP-labeled MT plus-ends in WT EB1-GFP-expressing MEFs plated over ICAM-1.** Images were collected over 5 minutes and captured at a rate of 2.98 frames/second. The video is representative of 3 independent experiments.
(WMV)

Video S5 **Time-lapse video showing movement of EB1-GFP-labeled MT plus-ends in mDia1^-/-^ EB1-GFP-expressing MEFs plated over ICAM-1.** Images were collected over 5 minutes and captured at a frame rate of 2.98 frames/second. The video is representative of 3 independent experiments.
(WMV)
