# Supplementary figures and images for: Mammalian Diaphanous-Related Formin 1 Regulates GSK3β-Dependent Microtubule Dynamics Required for T Cell Migratory Polarization
Source: PLoS One. 2013 Nov 18;8(11):e80500. doi: 10.1371/journal.pone.0080500 (PMC3832380; doi:10.1371/journal.pone.0080500)

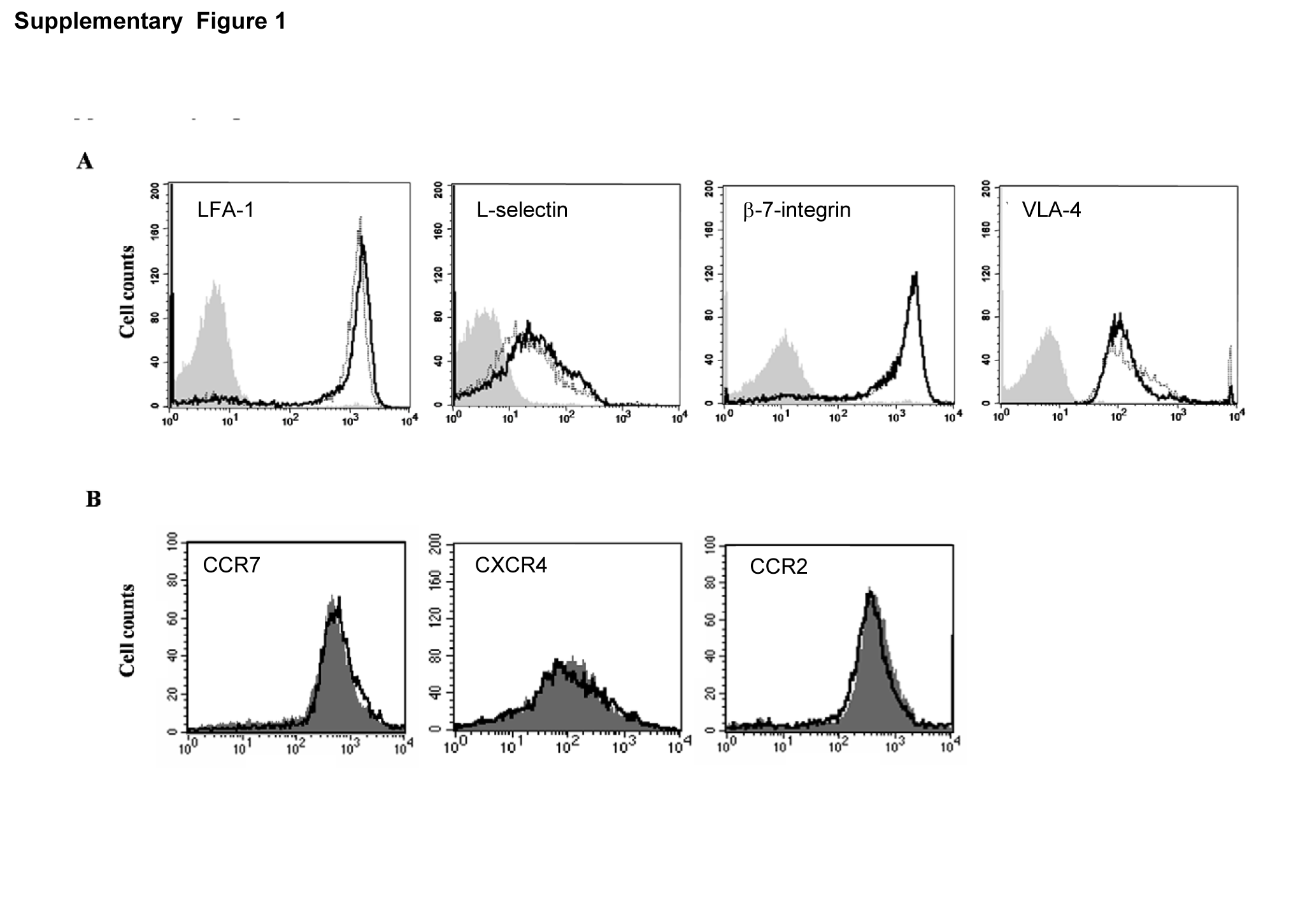

Supplement: Figure S1 — Expression of surface adhesion molecules and chemokine receptors in WT and mDia1-/- T cells. Histograms showing the expression of cell surface adhesion molecules and chemokine receptors on control and mDia1-/- T cells. Lymph node cells from mDia1-/- and wild-type (WT) mice were stained with the indicated fluorescently-labeled antibodies and analyzed by flow cytometry. (TIF) [file pone.0080500.s001.tif]

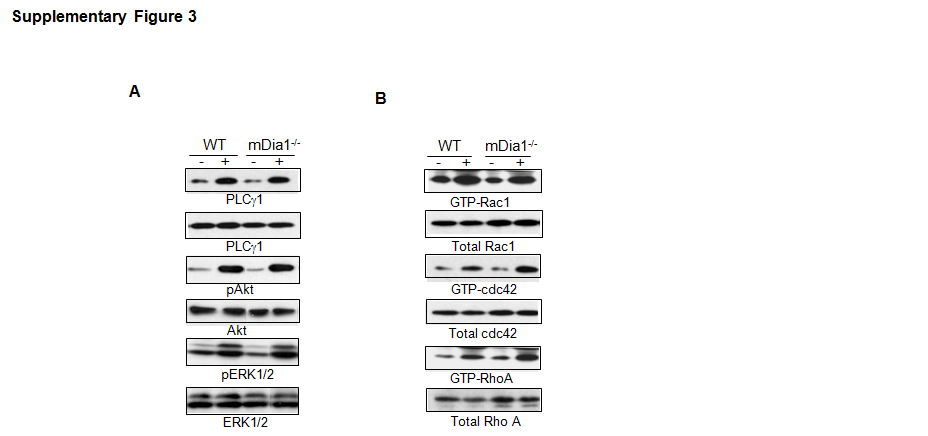

Supplement: Figure S3 — Analysis of mDia1 effects on proximal T cell signaling effectors. (A&B) mDia1-/- and WT T cells were left unstimulated or stimulated with CXCL12/ICAM-1 for 5 min and (A) the lysates then subjected to SDS-PAGE followed by immunoblotting with anti-phospho-PLCγ and anti-PLCγ antibodies, anti-phospho-Akt and then anti-Akt antibodies and anti-phospho-Erk1/Erk2 and then anti-Erk1/Erk2 antibodies; or (B) the lysates incubated with GST-rhotekin Rho-binding domain (to detect active Rho A) or GST-Pak1 protein-binding domain (to detect cdc42 or Rac1) fusion proteins immobilized on glutathione agarose beads and the precipitated proteins or whole cell lysates subjected to SDS-PAGE followed by immunoblotting with anti-Rac, cdc42 or RhoA antibodies. (TIF) [file pone.0080500.s003.tif]

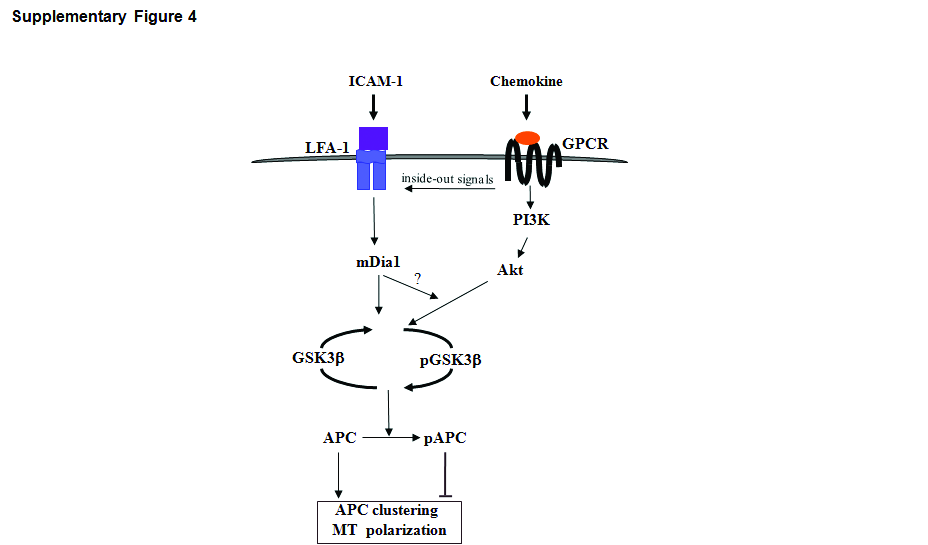

Supplement: Figure S4 — Schematic showing the proposed molecular pathway whereby mDia1 links LFA-1-engagement to MT stabilization and T cell polarization. Data from this study reveal involvement in linking LFA-1-ICAM-1 engagement in T cells to induction of GSK3β Ser/Thr phosphorylation and consequent inactivation. Because activated GSK3β normally evokes APC phosphorylation and degradation, mDia1-mediated GSK3β inactivation enables APC to accumulate at the MT plus-ends and thereby facilitate MT stabilization and polarization. By this means, mDia1 promotes LFA-1-mediated adhesion and T-cell transmigration and may enable LFA-1 to cooperate with chemokine-dependent directional cues to facilitate interstitial T cell migration. The mechanism whereby mDia1 modulates GSK3β phosphorylation is unknown, but appears to operate downstream or independently of Akt. pAPC: phosphorylated adenomatous polyposis coli; GPCR: G-protein-coupled receptor; pGSK3β: phosphorylated glycogen synthase kinase B. (TIF) [file pone.0080500.s004.tif]
